# Single basal application of thiacloprid for the integrated management of *Meloidogyne incognita* and *Bemisia tabaci* in tomato crops

**DOI:** 10.1038/srep41161

**Published:** 2017-01-25

**Authors:** Sa Dong, Xiaofen Ren, Dianli Zhang, Xiaoxue Ji, Kaiyun Wang, Kang Qiao

**Affiliations:** 1 Key Laboratory of Pesticide Toxicology & Application Technique, College of Plant Protection, Shandong Agricultural University, Tai’an, Shandong 271018, P.R. China; 2 College of Plant Protection, Nanjing Agricultural University, Nanjing, Jiangsu 210018, P.R. China; 3 Key Laboratory of Food Quality and Safety of Jiangsu Province, State Key Laboratory Breeding Base, Nanjing, Jiangsu 210018, P.R. China

## Abstract

Tomato growers commonly face heavy nematode (*Meloidogyne incognita*) and whitefly (B-biotype *Bemisia tabaci*) infestations, and previous studies demonstrated that thiacloprid could be used to control *M. incognita* and *B. tabaci* in cucumber. However, the efficacy of a single basal application of thiacloprid to control both pests and its effect on yield in tomato remains unknown. In this study, the potential of thiacloprid application to the soil for the integrated control of *M. incognita* and *B. tabaci* in tomato was evaluated in the laboratory and the field. Laboratory tests showed that thiacloprid was highly toxic to whitefly adults and eggs with an average lethal concentration 50 (LC_50_) of 14.7 and 62.2 mg ai L^−1^, respectively, and the LC_50_ of thiacloprid for nematode J2s and eggs averaged 36.2 and 70.4 mg ai L^−1^, respectively. In field trials, when thiacloprid was applied to the soil at 7.5, 15 and 30 kg ha^−1^ in two consecutive years, whitefly adults decreased by 37.8–75.4% within 60 days of treatment, and the root-galling index was reduced by 31.8–85.2%. Optimum tomato plant growth and maximum yields were observed in the 15 kg ha^−1^ treatment. The results indicated that a single basal application of thiacloprid could control *M. incognita* and *B. tabaci* and enhance tomato growth and yield.

Tomatoes (*Lycopersicon esculentum*) play an important role in vegetable production in China^1^, but in recent years, root-knot nematode disease and *tomato yellow leaf curl virus* (TYLCV) have become the key limiting factors in tomato production. The disease-causing agents proved to be the root-knot nematode (*Meloidogyne incognita*) and the vector whitefly (B-biotype *Bemisia tabaci*), respectively^2^,^3^.

*M. incognita* can infect nearly all roots of cultivated vegetables, making it perhaps the most common and destructive plant-parasitic nematode^4^, and it is pandemic in China, where it causes severe root-knot disease, especially in protected tomato cultivation^2^. *B. tabaci*, can pierce and suck sap from tomato plants, resulting in decreased yield and reduced fruit quality^5^, but even more seriously, *B. tabaci* can transfer a variety of plant viruses, such as TYLCV. In China, B-biotype *B. tabaci* has gradually replaced the indigenous whiteflies in most locations and has become a major insect pest in open-field and greenhouse production systems^6^. Traditional management is highly dependent on extensive use of soil fumigation to control *M. incognita* and insecticides to control *B. tabaci*, both of which are time-consuming and environmentally unfriendly, so efficient and reliable control measures are needed to limit reductions in tomato yields and prevent economic losses for producers.

Thiacloprid, [3-[(6-chloro-3-pyridinyl) methyl]-2-thiazolidinylidene] cyanamide, is a relatively new chloronicotinyl insecticide (Bayer AG, Germany)^7^ that exhibits good plant compatibility and has proven to be an excellent insecticide against sucking and biting insects^8^. It has been reported to be highly effective against *Caenorhabditis elegans*^9^ and Q-biotype *B. tabaci*^10^, while other chloronicotinyl insecticides (e.g., imidacloprid) have been widely applied to the soil to control aphids because of their outstanding systematic properties^11^. Our previous studies demonstrated that thiacloprid could be used in cucumber for the integrated control of *M. incognita* and *B. tabaci*^12^, so thiacloprid may be applied to the soil to control nematodes in tomato, and then be absorbed by the roots and transferred to the shoots to control whiteflies.

Therefore, the aims of the present study were to perform: (a) laboratory tests to determine the efficacy of thiacloprid in the control *M. incognita* and B-biotype *B. tabaci* and (b) field trials to evaluate its effects on *M. incognita* and B-biotype *B. tabaci* control and tomato crop productivity with a single basal soil application.

## Results

### Laboratory test

The lethal concentration required to kill 50% (LC_50_) (after 6 h) of adult B-biotype *B. tabaci* was calculated to be 14.7 mg ai L^−1^ for thiacloprid and 17.5 mg ai L^−1^ for imidacloprid. Accordingly, concentrations of 62.2 and 112.6 mg ai L^−1^ thiacloprid and imidacloprid, respectively, (after 6 d) were required to kill 50% of B-biotype *B. tabaci* eggs. The LC_50_ values of thiacloprid and abamectin for J2s of *M. incognita* were 36.2 and 8.51 mg ai L^−1^, respectively, while 70.4 and 0.61 mg ai L^−1^ were required to inhibit 50% of the *M. incognita* eggs during a 24 h exposure at 25 °C (Table 1).

### Field trials

The whitefly population was significantly reduced by different doses of thiacloprid in both years, 54.2–90.9% for 1 and 10 days after treatment for all doses. Although the efficacy declined a little on the 60^th^ day, it remained between 37.8–75.4%. The efficacy of the 7.5 kg ha^−1^ thiacloprid treatment (34.6–72.7%) was lower than the higher doses, but significantly higher than the abamectin treatments. The whitefly population in the abamectin treatment was not significantly different from the control, indicating that this chemical was ineffective against whiteflies (Table 2).

In this research, only *M. incognita* was isolated while other species of nematodes were below the detectable level. The same trend was observed in both years of the experiments: there was a positive relationship between nematode inhibition and thiacloprid dose. Thiacloprid at the dose of 30 kg ha^−1^ was the most effective for controlling nematodes, and it reduced the nematode population by 77.0% and 71.2% on the 60^th^ day in the two successive years, respectively. Additionally, the root galling index was reduced by 84.6% and 85.2% (Table 2). In the 15 kg ha^−1^ thiacloprid treatment, the root galling index was reduced by 78.5% and 81.9%, respectively, in 2011–2012 and 2012–2013, which was not significantly different from the 30 kg ha^−1^ treatment. The effect of 15 kg ha^−1^ of thiacloprid was comparable to abamectin at the dose of 7.5 L ha^−1^. The lowest dose of thiacloprid (7.5 kg ha^−1^) significantly decreased the number of nematodes and the root galling index relative to the control, but its effect was significantly lower than higher doses of thiacloprid and the abamectin treatments.

The heights of the tomato plants were increased by thiacloprid and abamectin in both years, and all thiacloprid treatments resulted in taller plants than the abamectin treatments (Table 3). The greatest plant heights in the two successive years were obtained in plots treated with 15 kg ha^−1^ thiacloprid (100.2 and 122.3 cm on 50 DAT, respectively); 16.9% and 14.6% greater than the control, respectively. It is noteworthy that there was not a positive relationship between plant height and thiacloprid dose in the experiment; where the intermediate dose (15 kg ha^−1^) resulted in the highest plant height. Tomato plant vigour followed a similar trend to the plant height in both seasons: the highest plant vigour was observed in plots treated with 15 kg ha^−1^ thiacloprid, followed by 30 kg ha^−1^ thiacloprid and abamectin, and then 7.5 kg ha^−1^ thiacloprid. Moreover, all treatments resulted in more favourable results than the untreated control.

In the 2011–2012 growing season, the highest yield was obtained with 15 kg ha^−1^ of thiacloprid treatment in the three fruit categories and the total marketable yield reached 90.1 t ha^−1^, which was higher than under abamectin but not significantly different (Table 4). The lowest yield was achieved in the untreated control with a reduction of 36.4% relative to 15 kg ha^−1^ of thiacloprid. Tomato yields with thiacloprid at doses of 7.5 and 30 kg ha^−1^ were significantly less than with abamectin and 15 kg ha^−1^ thiacloprid in all three categories and in total, but they were still much higher than the untreated control. The same trends in tomato fruit weight and total yield were observed in the 2012–2013 growing season, but the yields were generally higher than the previous year.

## Discussion

In China, *M. incognita* and B-biotype *B. tabaci* have become two of the most important agricultural pests, attacking a wide range of crops and causing dramatic yield losses^13^,^14^. Nematodes are mainly controlled by soil application of pesticides, and whitefly control relies on repeated foliar spraying. These practices are not only time intensive but they increase the risks to the health of the operators and environmental pollution, so more secure and less time-consuming control strategies are required.

Soil application could reduce the risk of pesticide poisoning and environmental pollution by lowering the impact of pesticides on the above-ground beneficial insects. Thiacloprid is considered to have the potential for the integrated control of both nematodes and whiteflies through soil application^12^. Laboratory studies showed that thiacloprid had a high potential to control the adults and eggs of B-biotype *B. tabaci*, better than that of imidacloprid. Moreover, it also had a certain nematicidal potential, although it was not as efficient as abamectin, a well-known and widely used nematicide^15^. Field trials indicated that a single basal soil application of thiacloprid was effective at reducing the infection of nematode and whitefly infestation, and the decrease in whiteflies in the thiacloprid treatments in this study may be attributable to the lethal action of the transfer of thiacloprid from the soil. In contrast, the result may have been due to a repellent effect, similar to that of another neonicotinoid insecticide imidacloprid, which was observed to possess antifeedant activity on aphids after seed treatment^16^. In this study, soil application of abamectin effectively controlled nematodes, but it was ineffective against whiteflies. This is probably because abamectin does not act systemically and could not be transferred to the above ground parts of the plants^17^.

Our results also demonstrate that applying thiacloprid to the soil could effectively control whiteflies for more than 60 days, which is similar to results with imidacloprid that reported a long persistence (45–60 days) when seeds were dressed to control aphids^18^,^19^. This may be because thiacloprid dissipates slower in the soil than on the plant^20^. Thiacloprid would be gradually released from the soil and absorbed into the roots, thus persisting longer as reported for imidacloprid^21^.

Our research also found that all treatments significantly enhanced tomato plant height, vigour and yield compared with the untreated control. Moreover, the use of thiacloprid benefited tomato yield structure and exhibited a stable performance in two field trials. This could be attributed to the control of nematodes and whiteflies by thiacloprid, which promoted tomato growth. On the other hand, this result might also be related to a series of biochemical reactions induced by thiacloprid in the plant^22^. Thiacloprid may promote the activities of leaf protective enzymes and eliminate the damage induced by free radicals; similar effects have been indicated with imidacloprid^23^. It also had been reported that neonicotinoids induce systemic acquired resistance (SAR) in plants, which would facilitate resistance against negative factors such as pathogens^24^. However, the dose of thiacloprid should be carefully selected because the best effect on plant growth was observed at the intermediate concentration, so slight phytotoxicity may emerge at higher doses. This is consistent with a report that the rates or frequency of imidacloprid application must be reduced to avoid phytotoxicity in grapefruit^24^.

In conclusion, the results of this study revealed that a single basal soil application of thiacloprid exhibited good integrated control efficacy against *M. incognita* and B-biotype *B. tabaci* and highly enhanced tomato plant growth. However, further studies that consider insecticide persistence, the growth promotion mechanism, and the optimal dosages and application times are necessary to achieve better tomato crop performance.

## Methods

### Chemicals

Thiacloprid (98.7% pure, Bayer CropScience, Greater China). Thiacloprid (36% pure, WG, Shandong United Pesticide Industry Co. Ltd., China). Imidacloprid (98.7% pure and 70% WP, Shandong United Pesticide Industry Co. Ltd., China). Abamectin (1.8% EC, Shandong United Pesticide Industry Co. Ltd., China).

### Laboratory test: thiacloprid toxicity against adult B-biotype *B. tabaci*

A number of *B. tabaci* B biotype were separated from a colony at Shandong Agricultural University (SDAU) Gardening Experiment Station, Tai’an, Shandong, China and reared on cabbage (*Brassica oleracea*) plants, variety Jingfeng No. 1, without any exposure to pesticides for 5 years. The stock culture was reared in a temperature-controlled greenhouse at 25 ± 2 °C.

Contact application was used to determine the toxicity of thiacloprid and imidacloprid against adult B-biotype *B. tabaci*^25^. Technical grade thiacloprid and imidacloprid were dissolved in acetone to various concentrations (100, 50, 25, 10, 5 and 1 mg ai L^−1^), and the pesticide solutions (0.5 mL) was then added to glass tubes. The tubes were rolled using a conventional hot dog roller until the acetone evaporated and the inner tube surfaces were evenly dried; the tubes were then placed in a ventilation hood for 2 h to fully dry. Approximately forty adult whiteflies were aspirated into each test tube, and then tubes were placed in an incubator at 25 ± 2 °C, 76 ± 5% RH. Mortality was determined after 6 h using a binocular microscope, and individuals were scored as dead when they were unable to right themselves after the tubes were tapped ten times on a countertop. Only 0.5 mL of acetone was added to the control tube. The tests were performed on the day of insecticide application, and all tests had three replications with four subsamples in each.

### Thiacloprid toxicity against B-biotype *B. tabaci* eggs

A small B-biotype *B. tabaci* culture was transferred weekly from the above-mentioned greenhouse and reared on three-week-old cotton plants (*Gossypium hirsutum* L), planted in 6.25 × 6.25 cm pots. The inoculated cotton plants were kept in insect-proof cages (60 × 60 × 60 cm) in an air-conditioned laboratory at 25 ± 1 °C, 76 ± 5% RH and a 12 : 12 h (L : D) photoperiod.

Cotton plants were trimmed so that only two true leaves were left and placed in the above-mentioned cages, where adult whiteflies were allowed to oviposit for approximately 24 h. After the adult insects were removed, the cotton plants were inspected to ensure that a minimum of 30 eggs had been posited. The leaves with eggs were dipped into the 100, 50, 25, 10, 5.0 and 1.0 mg ai L^−1^ thiacloprid and imidacloprid solutions (prepared in 5% acetone solution) for 5 s^26^, while controls were dipped into 5% acetone solution for 5 s. The tests were replicated three times with four subsamples in each replication. Plants were moved to a growth chamber and cultured under 25 ± 2 °C, 76 ± 5% RH and a 14 : 10 h (L : D) photoperiod. Evaluations were made after 6 days by counting the dead (shrivelled) and live eggs and nymphs present on the underside of the leaves.

### Thiacloprid toxicity against J2 *M. incognita*

The experimental population of *M. incognita* was originally isolated from tomato plants in central Shandong Province and maintained on tomato roots in the SDAU greenhouse. Perineal configuration, esterase electrophoretic pattern and host range analyses were applied to classify the isolate. For the experiments, all plants were maintained in the greenhouse at 25 ± 2 °C, 60% RH and a 16 : 8 h (L : D) photoperiod in plastic pots (20 cm in diameter), and eight-week-old tomato plants were used for the inoculations. After one and a half months, the tomato plants were uprooted, and the roots were washed free of soil and cut into 2 cm pieces. Eggs were extracted according to the NaOCl procedure; briefly, *M. incognita* egg masses were collected with forceps, rinsed with sterile water, placed in 0.5% NaOCl solution agitated for 4 minutes and then rinsed with sterile water on a 26-μm sieve. Second-stage juveniles (J2s) were allowed to hatch in modified Baermann funnels at 25 °C^27^; J2s that hatched in the first 3 days were discarded, and only those collected after another 24 h were used in the experiments.

An aqueous test was used to confirm the nematicidal potential of thiacloprid and abamectin using J2s of *M. incognita*. A series of thiacloprid and abamectin doses (1.0, 5.0, 10, 25, 50, 100 and 200 mg ai L^−1^) were prepared in acetone + sterile distilled water (10:90% by volume), and both sterile distilled water and a mixture of water with acetone (concentrations equivalent to those in the treatments) were set as controls. Then 1 mL of both solution and approximately 150 root-knot nematode J2s were added to individual well of a 24-well plate, then wrapped with parafilm, and stored in aluminum foil pans covered with another pan to keep the units in darkness at 25 ± 2 °C. After 48 h, an inverted microscope (Olympus, China) was used to determine the relative percentages of the motile and immotile J2s at 40x magnification and after washing in tap water over a 20 μm pore screen, all J2s were transferred to plain water to eliminate excess chemicals. Additionally, 30 immobile J2 larvae were collected from the above experiments, transferred to other tissue culture plates filled with water, and monitored for 12 h to confirm the nematicidal activity of both chemicals. All experiments followed a completely random design, with five replicates and were repeated three times.

### Thiacloprid toxicity against *M. incognita* eggs

Egg masses of *M. incognita* were picked from inoculated tomato roots, and the eggs were extracted using the method described by Hussey and Barker^27^. For the egg-hatching experiments, thiacloprid and abamectin treatments (200, 100, 50, 25, 10, 5 and 1 mg ai L^−1^) were prepared in 5% acetone solution, and 1 mL of solution was added to the individual well of a 24-well plate and inoculated with 10 μL of egg suspension which contained approximately 100 eggs. Controls were prepared in 1 mL 5% acetone solution. Plates were placed at 25 °C for 24 h, and the eggs were then washed free of the chemicals and incubated for 6 days in clean water. Hatchability was recorded by observing the eggs at 40x magnification and evaluating the relative hatching percentages. The tests were replicated three times with four subsamples in each replicate.

### Field trials: site description and treatment application

Field trials were conducted in a commercial tomato field near Tai’an City, Shandong Province, China, in two successive cropping seasons during 2011–2012 and 2012–2013. All experimental protocols were approved by the owners of the farm. The farm had been in conventional tomato production for 10 years before the start of the experiment, and the soil was a silt loam with an organic matter content of 19.0–24.3 g kg^−1^ soil, a pH of 7.0–7.2 and a bulk density of approximately 1.2 g cm^−3^. The soil was disked twice, and the beds were spaced at 0.7 m apart center to center (0.8 m wide × 0.2 m high).

Five treatments were applied with three replications as follows: (a) abamectin (1.8% EC, AI) furrow applied at a dose of 7.5 L ha^−1^, (b) thiacloprid (36% WG, AI) furrow applied at a dose of 7.5 kg ha^−1^, (c) thiacloprid furrow applied at a dose of 15 kg ha^−1^, (d) thiacloprid furrow applied at a dose of 30 kg ha^−1^, and (e) an untreated control. Local farmers typically carry out traditional solarization and then furrow apply abamectin rather than any other pesticides, so abamectin was tested as a reference treatment in this experiment.

Individual 20-m^2^ plot consisting of four neighbouring rows was laid out in a randomized block design. On the day of treatment (August 11, 2011 and August 15, 2012), thiacloprid and abamectin were furrow applied to the soil at a depth of 0.20 m in only the planting rows. The planting rows were then bedded, and 20 six-week-old ‘Jinpengwuxian’ tomato seedlings (Xinyuan Seedling Co., Ltd., Shandong, China) were transplanted into the top of the beds at a row spacing of 0.50 m. Plants were staked and tied as needed, and ordinary flood irrigation was provided according to the water requirements of the crops.

### Whitefly infestation assays

Five tomato plants were selected randomly from each plot at 1, 10, 20, 40 and 60 days intervals after treatments. *B. tabaci* adults on the underside of the leaves were carefully quantified by turning up the leaf in the early morning when the insects common rest in groups.

### Nematode infestation assays

Soil samples were collected with a soil probe (2.5 cm wide by 20 cm deep) from the rhizosphere of ten tomato plants per plot at 20, 40 and 60 days after treatment (DAT). The nematodes extracted from 100 cm^3^ soil samples were counted using a standard sieving and centrifugation procedure^28^, and the root galling index was determined at 14 weeks after treatment (WAT) by digging the roots of five plants per plot and evaluating root damage on a scale of 0–10, where 0 = no galls, 1 = 0–10% and 10 = 90–100% of roots galled^29^.

### Plant growth and yield assays

The plant heights of ten plants were averaged as measured from the soil line to the tip of the growing terminal by the same person at 30 and 50 DAT. Plant vigour was visually assessed at 8 WAT using a percentage scale, where 0% = plant death and 100% = optimum growth. All data were collected by the same person to ensure standardization. Tomato fruits were harvested twice (12 and 14 WAT), which is a typical practice in greenhouses in the northern China. The marketable tomatoes were graded according to current market standards of extra-large, large and medium and the total weights were evaluated.

### Statistical analysis

Data from the laboratory tests were analysed by logit/probit dose response/mortality regression calculated using the SPSS probit procedure (SPSS, version 17.0 for Windows, IBM, Chicago, IL). Adjusted mortality was calculated using Schneider-Orelli’s formula, whereby mortality was calculated as a percentage and adjusted to mortality in the control (solvent only) using the equation:


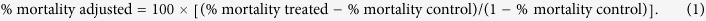


Data were expressed as percentages and arcsine transformed to homogenize the variances before analysis. Analysis of variance (ANOVA) was carried out on the control effects of different treatments. Treatment means was compared by the Student-Newman-Keuls test (SPSS, version 17.0 for Windows). Differences between values at *P* < 0.05 were considered statistically significant.

## Additional Information

**How to cite this article**: Dong, S. *et al*. Single basal application of thiacloprid for the integrated management of *Meloidogyne incognita* and *Bemisia tabaci* in tomato crops. *Sci. Rep.* **7**, 41161; doi: 10.1038/srep41161 (2017).

**Publisher's note:** Springer Nature remains neutral with regard to jurisdictional claims in published maps and institutional affiliations.

## Figures and Tables

**Table 1:** Toxicity of chemicals to adults and eggs of *Bemisia tabaci* B biotype and *Meloidogyne incognita.*

Chemicals	Slope ± SE	LC_50_ (mg L^−1^)^a^	R^2^
***Bemisia tabaci*****B biotype**			
Adult			
Thiacloprid	1.48 ± 0.56	14.7 (6.8–21.8)^b^	0.96
Imidacloprid	1.06 ± 0.57	17.5 (8.2–34.2)	0.97
Eggs			
Thiacloprid	2.66 ± 0.38	62.2 (45.5–76.6)	0.98
Imidacloprid	2.34 ± 0.32	112.6 (51.1–173.1)	0.96
***Meloidogyne incognita***			
J2			
Thiacloprid	1.47 ± 0.17	36.2 (23.8–48.7)	0.97
Abamectin	1.47 ± 0.12	8.51 (6.20–10.92)	0.96
Eggs			
Thiacloprid	1.99 ± 0.28	70.4 (52.8–85.6)	0.94
Abamectin	1.70 ± 0.67	0.61 (0.03–0.97)	0.99

^a^Data are arithmetic means of three replications and calculated with SPSS.

^b^95% Fiducial limits (from probit analysis).

**Table 2:** Effect of thiacloprid (36% WG) and abamectin (1.8% EC) against whiteflies and nematodes in the consecutive two years’ field trials.

Treatments	Dose per ha	Whiteflies in 5 tomato plants^a^					Nematodes in 100 cm^3^ soil^b^			Root galling index^c^	Nematode control effect (%)^d^
		1 DAT	10 DAT	20 DAT	40 DAT	60 DAT	20DAT	40DAT	60DAT		
**2011–2012 experiment**											
Thiacloprid	30 kg	2.0 ± 0.6c	6.0 ± 0.6c	14 ± 3.1c	11 ± 1.0c	18 ± 1.0c	2.7 ± 0.9b^e^	5.3 ± 1.7b	6.7 ± 1.5c	0.67 ± 0.0c	84.6a
Thiacloprid	15 kg	3.0 ± 0.6c	5.0 ± 0.0c	18 ± 2.5c	16 ± 1.0c	23 ± 1.7c	4.0 ± 1.0b	7.0 ± 0.6b	9.0 ± 0.6bc	0.92 ± 0.0c	78.5a
Thiacloprid	7.5 kg	6.0 ± 1.0b	13 ± 0.6b	22 ± 3.1bc	28 ± 1.5b	28 ± 4.6bc	6.0 ± 1.5ab	7.7 ± 1.5b	13.7 ± 2.2b	2.92 ± 0.8b	31.8b
Abamectin	7.5 L	23 ± 0.6a	45 ± 2.6 a	33 ± 1.5ab	35 ± 1.5b	38 ± 5.2ab	3.0 ± 0.6b	6.3 ± 0.3b	7.3 ± 0.9c	0.78 ± 0.5c	81.8a
Control	—	22 ± 0.6a	43 ± 2.1 a	39 ± 3.8a	46 ± 4.6a	45 ± 3.8a	9.7 ± 1.8a	20.7 ± 2.3a	28.7 ± 2.0a	4.28 ± 0.2a	—
**2012–2013 experiment**											
Thiacloprid	30 kg	2.0 ± 0.0c	3.0 ± 0.6c	10 ± 0.6c	12 ± 0.6c	15 ± 0.6d	2.0 ± 0.6b	5.3 ± 0.3c	6.0 ± 0.6d	0.59 ± 0.1c	85.2a
Thiacloprid	15 kg	2.0 ± 0.6c	5.0 ± 0.6c	14 ± 2.5c	11 ± 0.6c	21 ± 4.5cd	3.3 ± 0.3b	6.7 ± 0.3c	8.7 ± 0.3c	0.72 ± 0.0c	81.9a
Thiacloprid	7.5 kg	5.0 ± 1.0b	11 ± 0.6b	24 ± 3.1b	34 ± 2.5b	29 ± 1.2c	7.3 ± 1.8a	9.7 ± 0.9b	12.3 ± 0.3b	2.56 ± 0.2b	83.7a
Abamectin	7.5 L	13 ± 0.6a	20 ± 2.1a	38 ± 3.8a	41 ± 6.1b	42 ± 1.5b	2.7 ± 0.3b	6.0 ± 0.6c	8.3 ± 0.3c	0.65 ± 0.1c	35.7b
Control	—	14 ± 1.0a	24 ± 2.1a	41 ± 3.2a	52 ± 3.8a	61 ± 4.6a	8.7 ± 0.9a	16.0 ± 1.2a	20.3 ± 0.3a	3.98 ± 0.4a	—

^a^Whiteflies (*Bemisia tabaci* B biotype) were counted at 1, 10, 20, 40 and 60 DAT (days after treatment) in two growing seasons. Data are arithmetic means of three replications and means separated with Student-Newman-Keuls test (*P* < 0.05). Numbers in the same column followed by the same letter are not significantly different according to Student-Newman-Keuls test (*P* < 0.05).

^b^Nematodes [*Meloidogyne incognita* (Kofoid & White) Chitwood] in 100 cm^3^ soil were counted at 20, 40 and 60 DAT in two growing seasons.

^c^Nematode root galling index was determined at 14 WAT (weeks after treatment) using a 0–10 scale where 0 = no galls and 10 = 100% of roots galled.

^d^Reduction of root galling index relative to control.

^e^Data are arithmetic means of three replications and means separated with Student-Newman-Keuls test (*P* < 0.05). Numbers in the same column followed by the same letter are not significantly different according to Student-Newman-Keuls test (*P* < 0.05).

**Table 3:** Effect of thiacloprid (36% WG) and abamectin (1.8% EC) on tomato plant height and vigor in the consecutive two years’ field trials.

Treatments	Dose per ha	Plant height^a^ (cm)		Plant vigor^b^
		30 DAT	50 DAT	
**2011–2012 experiment**				
Thiacloprid	30 kg	45.6 ± 1.9^c^ab	96.4 ± 0.3ab	85 ± 2.6ab
Thiacloprid	15 kg	49.7 ± 1.1a	100.2 ± 3.1a	91 ± 0.9a
Thiacloprid	7.5 kg	43.3 ± 2.0ab	93.7 ± 1.9ab	84 ± 1.9ab
Abamectin	7.5 L	42.7 ± 1.5ab	91.8 ± 1.9b	86 ± 1.6ab
Control	—	39.6 ± 1.6b	85.7 ± 0.9c	79 ± 1.4b
**2012–2013 experiment**				
Thiacloprid	30 kg	56.4 ± 1.1ab	119.1 ± 1.2a	88 ± 1.2ab
Thiacloprid	15 kg	58.1 ± 0.6a	122.3 ± 0.9a	92 ± 0.9a
Thiacloprid	7.5 kg	53.6 ± 1.5ab	115.4 ± 0.4b	85 ± 2.3bc
Abamectin	7.5 L	52.1 ± 2.2bc	113.3 ± 1.7b	87 ± 2.2ab
Control	—	48.7 ± 0.7c	106.7 ± 0.2c	80 ± 1.0c

^a^Plant height was determined at 30 and 50 DAT (days after treatment) in two growing seasons.

^b^Plant vigor was determined at 8 WAT (weeks after treatment), using a 0–100% scale where 0% = plant death and 100% = optimum growth.

^c^Data are arithmetic means of three replications and means separated with Student-Newman-Keuls test (*P* < 0.05).

**Table 4:** Effect of thiacloprid (36% WG) and abamectin (1.8% EC) on tomato marketable yields in the consecutive two years’ field trials.

Treatments	Dose per ha	Extra-large (t ha^−1^)	Large (t ha^−1^)	Medium (t ha^−1^)	Marketable (t ha^−1^)	Yield increase (%)
**2011–2012 experiment**						
Thiacloprid	30 kg	5.6 ± 0.2b^a^	17.5 ± 0.7b	47.2 ± 3.0bc	70.3 ± 3.0b	22.7
Thiacloprid	15 kg	6.7 ± 0.6a	25.9 ± 1.6a	57.5 ± 3.6a	90.1 ± 5.4a	57.2
Thiacloprid	7.5 kg	5.3 ± 0.3b	17.2 ± 0.8b	44.6 ± 0.3bc	67.1 ± 1.1bc	17.1
Abamectin	7.5 L	6.4 ± 0.3ab	24.2 ± 1.5a	52.7 ± 1.2ab	83.3 ± 2.3a	45.4
Control	—	4.0 ± 0.1c	14.8 ± 2.3b	38.5 ± 2.8c	57.3 ± 3.8c	—
**2012–2013 experiment**						
Thiacloprid	30 kg	11.7 ± 0.4b	25.1 ± 1.3b	57.5 ± 2.7b	94.3 ± 3.8c	30.8
Thiacloprid	15 kg	15.2 ± 0.7a	30.2 ± 0.7a	75.7 ± 1.2a	121.1 ± 1.4a	68.0
Thiacloprid	7.5 kg	10.5 ± 0.5b	23.5 ± 0.8b	55.6 ± 1.7b	89.6 ± 2.9c	24.3
Abamectin	7.5 L	13.9 ± 0.2a	28.6 ± 1.6a	68.5 ± 1.4a	111.0 ± 2.3b	54.0
Control	—	8.1 ± 0.1c	16.7 ± 0.1c	47.3 ± 4.2c	72.1 ± 4.0d	—

^a^Data are arithmetic means of three replications and means separated with Student-Newman-Keuls test (*P* < 0.05). Numbers in the same column followed by the same letter are not significantly different according to Student-Newman-Keuls test (*P* < 0.05).
